# Development and Validation of a Weather-Based Model for Predicting Infection of Loquat Fruit by *Fusicladium eriobotryae*

**DOI:** 10.1371/journal.pone.0107547

**Published:** 2014-09-18

**Authors:** Elisa González-Domínguez, Josep Armengol, Vittorio Rossi

**Affiliations:** 1 Instituto Agroforestal Mediterráneo, Universidad Politécnica de Valencia, Valencia, Spain; 2 Istituto di Entomologia e Patologia vegetale, Università Cattolica del Sacro Cuore, Piacenza, Italy; Institute of Plant Physiology and Ecology, China

## Abstract

A mechanistic, dynamic model was developed to predict infection of loquat fruit by conidia of *Fusicladium eriobotryae*, the causal agent of loquat scab. The model simulates scab infection periods and their severity through the sub-processes of spore dispersal, infection, and latency (i.e., the state variables); change from one state to the following one depends on environmental conditions and on processes described by mathematical equations. Equations were developed using published data on *F. eriobotryae* mycelium growth, conidial germination, infection, and conidial dispersion pattern. The model was then validated by comparing model output with three independent data sets. The model accurately predicts the occurrence and severity of infection periods as well as the progress of loquat scab incidence on fruit (with concordance correlation coefficients >0.95). Model output agreed with expert assessment of the disease severity in seven loquat-growing seasons. Use of the model for scheduling fungicide applications in loquat orchards may help optimise scab management and reduce fungicide applications.

## Introduction

Scab, caused by the plant-pathogenic fungus *Fusicladium eriobotryae* (Cavara) Sacc., is the main disease affecting loquat in Spain and in the whole Mediterranean basin [1], [2]. The fungus affects young twigs, leaves and fruits, causing circular olive-colored spots that, on fruits, reduce their commercial value [1]. *Fusicladium* spp. are the anamorphic stages of the ascomycete genus *Venturia* but the sexual stage of *F. eriobotryae* has never been found in nature [2].

Although loquat scab is a well-known problem in the areas where loquat trees are cultivated, the biology of *F. eriobotryae* and the epidemiology of the disease have been seldom studied [1], [3]–[8]. These studies have depicted *F. eriobotryae* as a highly rain-dependent pathogen that requires mild temperatures and long wet periods to infect loquat trees.

Environmental requirements for infection and the dispersion patterns have been studied in detail for other *Venturia* spp., such as *Venturia inaequalis* [9]–[16], *V. nashicola* [17]–[19], *V. pyrina* [20]–[24], *F. carpophilum* [25], [26], *F. effusum* [27]–[29], and *F. oleagineum* [30]–[35]. These studies have been used to elaborate epidemiological models for some of these pathogens including *V. pyrina* [36], *V. nashicola* [37], *V. inaequalis* [38], [39], *F. oleagineum* [40], and *F. effusum* [41]. For *V. inaequalis*, the use of epidemiological models to schedule fungicide applications has reduced the number of treatments [42]–[45]. To date, no epidemiological model has been developed for *F. eriobotryae*.

Disease modelling is an important step towards the implementation of sustainable agriculture [46], [47]. Since the 1990 s, modern crop production has focused on the implementation of less intensive systems with reduced inputs of fertilizers and pesticides, and reduced use of natural resources [46]. Sustainable agriculture has its roots in Integrated Pest Management (IPM) [48]. IPM concepts originated as a reaction to the disruption of agro-ecosystems caused by massive applications of broad-spectrum pesticides in the middle of the last century [46] and also because of concern about the effects of excessive pesticide use on human health [49].

In Europe, the implementation of IPM has been legislatively mandated in recent years because of Directive 2009/128/CE regarding sustainable use of pesticides. Among other actions, the Directive encourages EU Member States to promote low pesticide-input pest control and the implementation of tools for pest monitoring and decision making, as well as advisory services (Art. 14 of the Directive). De facto, the “sustainable use” directive has made IPM mandatory in European agriculture as of 2014. As a consequence, there is an increased interest in the development and use of plant disease models to improve the timing of pesticide applications and to thus limit unnecessary treatments [46], [50], [51].

Our aims in this paper were (i) to develop a mechanistic, dynamic model to predict infection of loquat fruit by the scab fungus *F. eriobotryae*, and (ii) to evaluate the model against three independent data sets. The model was elaborated based on the principles of “systems analysis” [52], [53] and by using recent data on the biology and epidemiology of *F. eriobotryae* obtained under environmentally controlled and field conditions [1], [6], [7].

## Model Development

**Figure 1:**
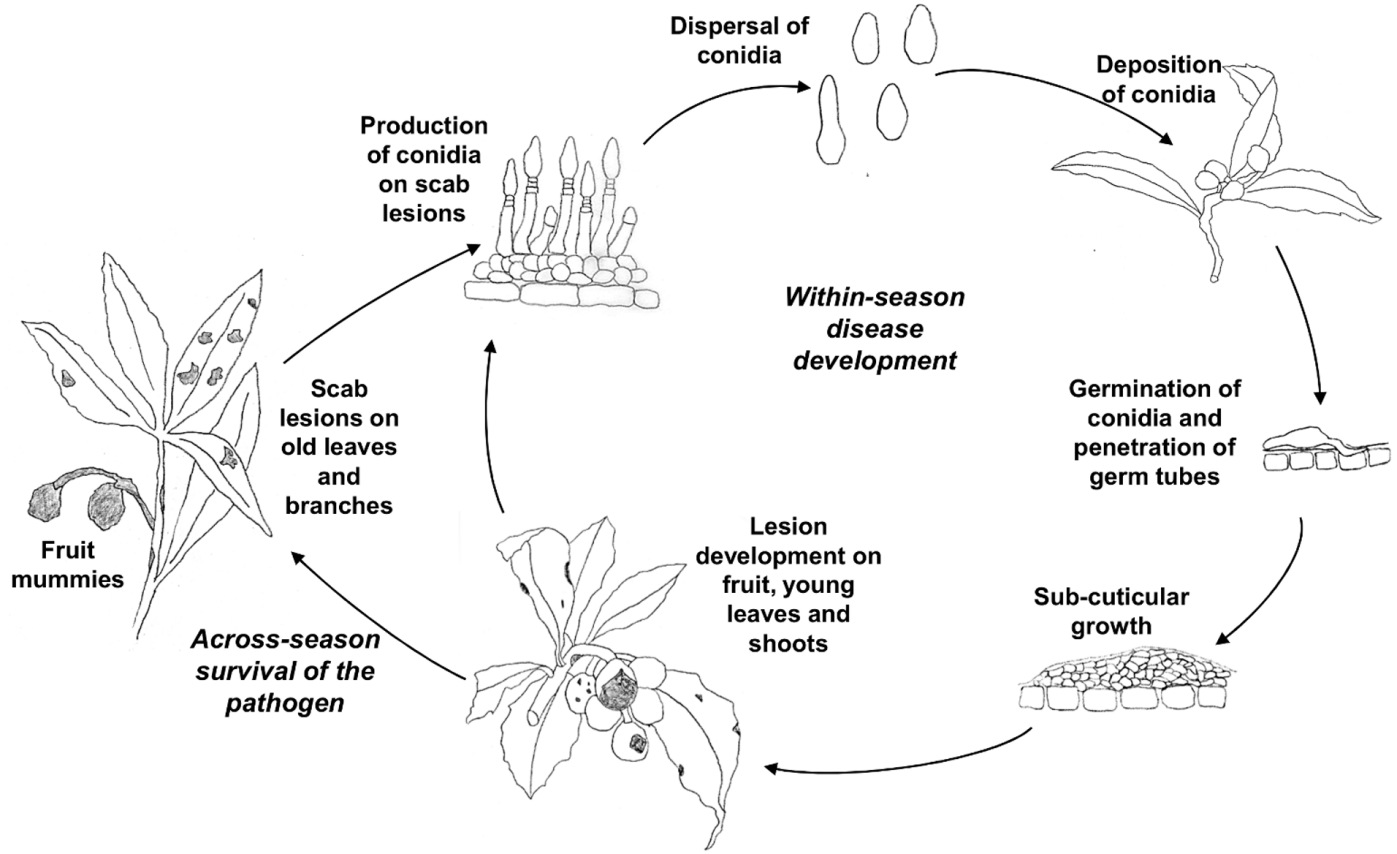
Disease cycle of loquat scab caused by *Fusicladium eriobotryae*.

Based on the available information [1], [3]–[8], the life cycle of *F. eriobotryae* under the Mediterranean climate is described in Figure 1. The fungus oversummers in lesions on branches and leaves and on mummified fruits that remain in the tree after harvest; during summer, high temperatures and low humidity may prevent sporulation on these lesions. Under favorable conditions in the fall, the conidia produced by the oversummering lesions serve as the primary inoculum and infect young leaves or loquat fruits. Conidia are dispersed by splashing rain to nearby fruits and leaves; with suitable temperature and wetness, conidia germinate and penetrate the tissue, probably directly through the cuticle or through stomata. Once infection has occurred and if the temperature is favorable, the fungus grows under the cuticle; conidiophores then erupt through the cuticle and produce new conidia. These conidia cause secondary infections during the entire fruiting season as long as rains disperse them and as long as temperature and wetness duration permit conidial germination, infection, and lesion growth.

### Model description

**Figure 2:**
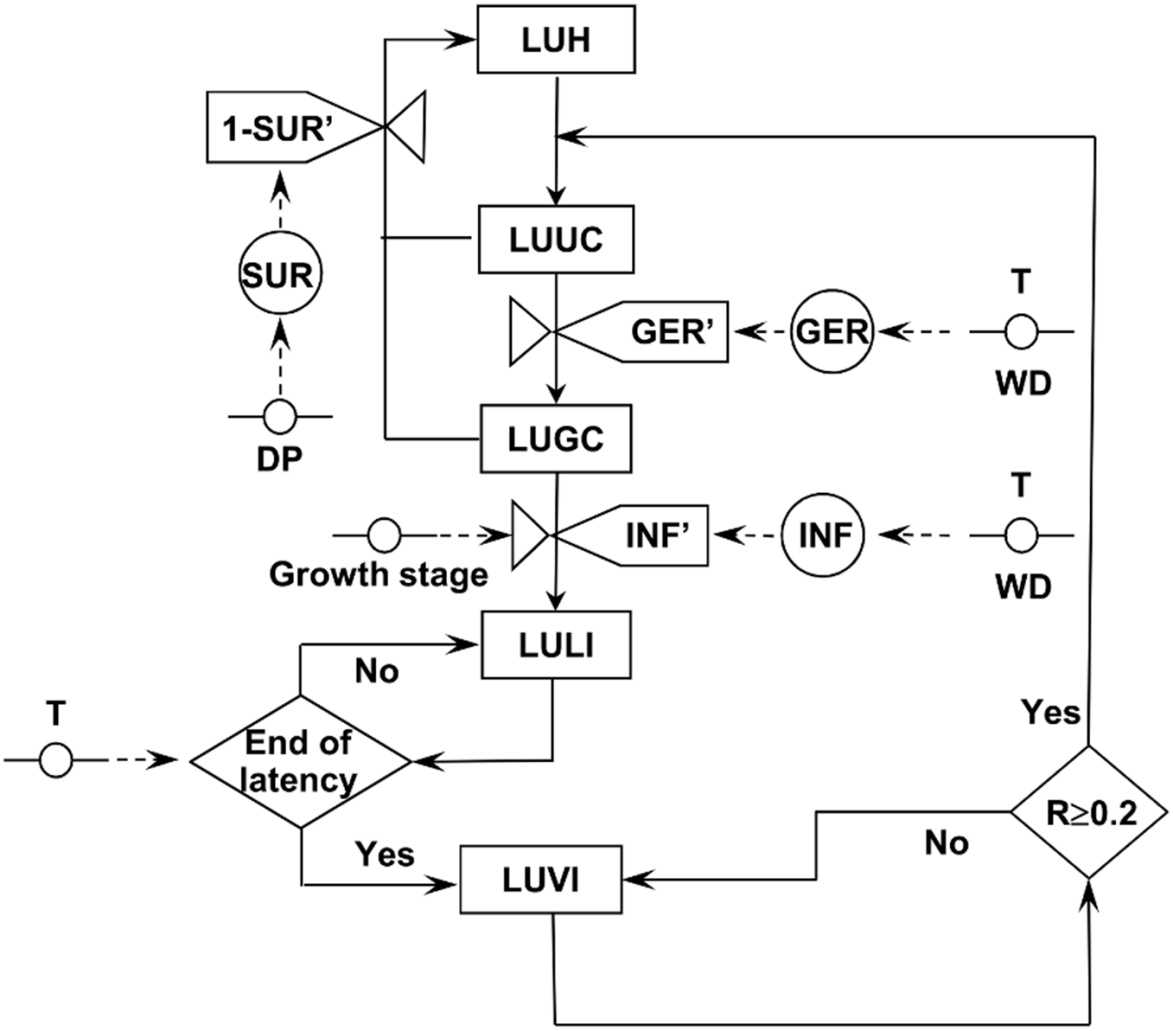
Relational diagram showing how the model simulates infection by *Fusicladium eriobotryae*. Legend: boxes are state variables; line arrows show fluxes and direction of changes from a state variable to the next one; valves define rates regulating these fluxes; diamonds show switches (i.e., conditions that open or close a flux); circles crossed by a line show parameters and external variables; dotted arrows show fluxes and direction of information from external variables or parameters to rates or intermediate variables; circles are intermediate variables. See Table 1 for acronym explanation.

The relational diagram of the model for loquat fruit infection by *F. eriobotryae* is shown in Figure 2, and the acronyms are explained in Table 1. The time step of the model is 1 hour.

**Table 1:** List of variables used in the model.

Acronym	Description	Unit
*T*	Air temperature	°C
*RH*	Relative humidity	%
*R*	Rainfall	mm
*VPD*	Vapour pressure deficit	hPa
*WD*	Wetness duration	hours
*Teq*	Temperature equivalent	°C
*LUH*	Unit of loquat fruit surface without conidia of *F. eriobotryae*	Number (0–1)
*LUUC*	Unit of loquat fruit surface with ungerminated conidia of *F. eriobotryae*	Number (0–1)
*LUGC*	Unit of loquat fruit surface with germinated conidia of *F. eriobotryae*	Number (0–1)
*LULI*	Unit of loquat fruit surface with latent infection by *F. eriobotryae*	Number (0–1)
*LUVI*	Unit of loquat fruit surface with visible scab lesions	Number (0–1)
*GER*	Cumulated conidial germination	Number (0–1)
*INF*	Cumulated infection	Number (0–1)
*SUR*	Cumulated conidial survival	Number (0–1)
*GER* **′**	Germination rate (first derivative of GERM)	Number (0–1)
*INF* **′**	Infection rate (first derivative of INF)	Number (0–1)
*SUR* **′**	Survival rate (first derivative of SUR)	Number (0–1)
*C*	Correction factor	Number (0–1)
*DD*	Degree days	Number

The model starts at fruit set and ends at harvest because fruits are assumed to be always susceptible to infection. The model considers the lesions from the previous season on branches, old leaves, and mummified fruits as the sources of primary inoculum. Because the abundance of these lesions in an orchard may vary depending on several conditions–on, for instance, the level of disease or the fungicide treatments in the previous season–and because it is difficult to quantify these lesions, the model assumes that oversummered forms are present in the orchard and that they hold conidia at fruit set and onwards.

The model considers that any measurable rain (i.e., R≥0.2 mm in 1 hour) causes dispersal and deposition of conidia on loquat fruit [7] and triggers an infection process that potentially ends with the appearance of scab symptoms. Each site on the fruit that is occupied by a conidium or conidia is considered a potential infection site and is referred to as a lesion unit (LU). During the infection process, infection on any LU can fail because conidia may fail to germinate or may germinate but then die because of unfavorable conditions. Therefore, the proportion of LUs that become scabbed at the end of the infection process may be less than that occupied by splashing conidia at the beginning of the process.

The model predicts the progress of infection on single LUs, which are the surface unit of the fruit which can become occupied by a scab lesion. This approach is related to the concept of “carrying capacity”. In ecology, the carrying capacity is interpreted broadly as the maximum population size that any area of land or water can sustain [54], [55]. In plant pathology, the host’s carrying capacity for disease is the maximum possible number of lesions that a plant (or an organ) can hold [56]. The carrying capacity is a common concept in plant disease modeling [57]–[60]. In the model described here, a LU is initially healthy (LUH) but then becomes occupied by: ungerminated conidia (LUUC) at the time of conidial dispersal; germinated conidia (LUGC) at the time of conidial germination; latent infection after penetration (i.e., hyphae are invading the fruit cuticle; LULI); and visible and sporulating scab lesions at the end of latency (LUVI). Both LUUC and LUGC can fail to progress if ungerminated or germinated conidia die; these LUs then return to being LUHs because they can start a new infection process whenever new conidia are splashed on them.

At any dispersal event on hour h, the model considers that LUUC_h_ = 1. The rate at which LUUC_h_ advances to LUGC_h_ depends on a germination rate (GER′), and the rate at which LUGC_h_ advances to LULI_h_ depends on an infection rate (INF′) (Figure 2). Both GER′ and INF′ are influenced by temperature (T in °C) and wetness duration (WD, in hours) (i.e., free water on the surface of the loquat fruit) caused by either rain or dew. Fruit surfaces are assumed to be wet on any hour when R_h_>0 mm, or RH_h_>89%, or VPD_h_<1, where VPD is the vapour pressure deficit (in hPa) calculated using T_h_ and RH_h_, following Buck [61]. The rate at which LUUC_h_ and LUGC_h_ returns to LUH_h_ depends on a survival rate (SUR′), which depends in turn on the length of the dry period (DP), i.e., the number of hours with no wetness on the fruit surface (Figure 2).

GER′, INF′, and SUR′ are calculated at hourly intervals by using the first derivative of the equations described in González-Domínguez et al. [6] in the form:
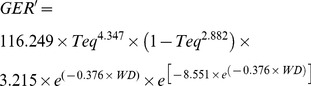
(1)

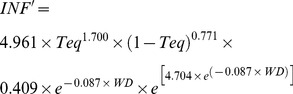
(2)

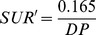
(3)where: *Teq* is the temperature equivalent in the form 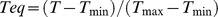 where: *T* is the temperature regime, *T_min_* = 0°C and *T_max_* = 35°C in equation (1), and *T_min_* = 0°C and *T_max_* = 25°C in equation (2); *WD* = number of consecutive hours with wetness; *DP* = number of consecutive hours with no wetness. When *DP* = 0, *SUR*′ = 1

At any time of the infection progress (*i*):
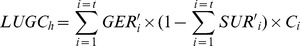


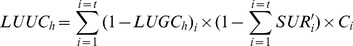


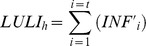


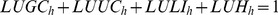
where *C* is a correction factor 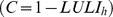
_._

**Figure 3:**
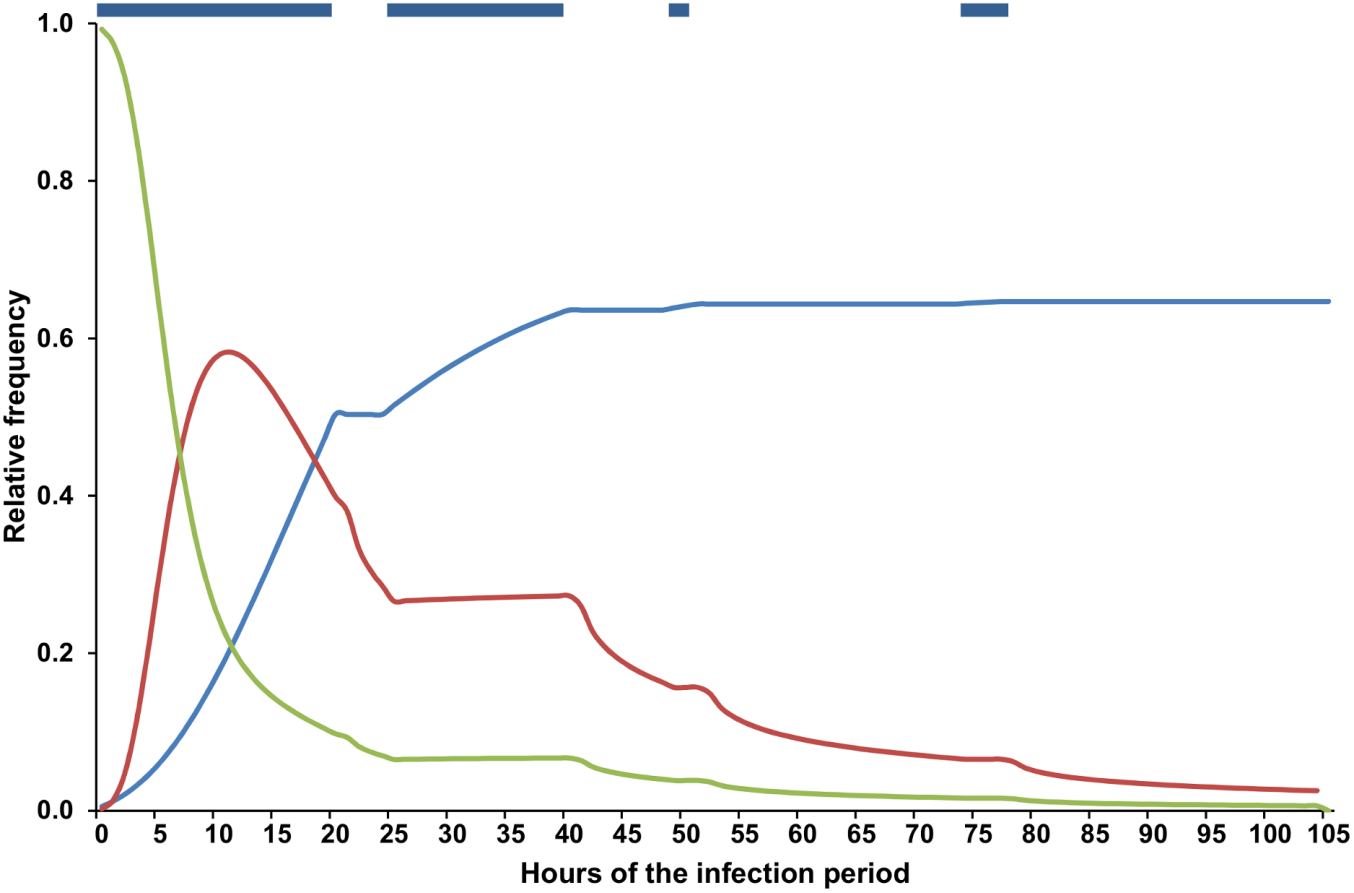
Dynamics of lesion units (LUs) during an infection period of *Fusicladium eriobotryae*. The graph shows the relative frequency of LUs occupied by ungerminated conidia (LUUC, in green), germinated conidia (LUGC, in red), and latent infections (LULI in blue). Blue bars at the top indicate hours with free water on the fruit surface. An infection period starts when a rain event splashes conidia on LUs and ends when no viable conidia are present on any LUs, i.e., when LUUC≤0.01.

Any infection period triggered by a conidial dispersal event ends when no viable conidia are present on any LUs, exactly when *LUUC*≤0.01. An example of model output for a single infection period is shown in Figure 3.

The model considers that any further rain event causes further dispersal and deposition of conidia if >5 hours have passed after the previous dispersal event. This is the time required by a lesion to produce new conidia.

### Model output

The model output consists of: (i) the available inoculum on fruits (i.e., the frequency of LUs with ungerminated conidia on each day) as a measure of the potential for infection to occur; (ii) the dynamics of *LULI* for each infection process; and (iii) the seasonal dynamics of the accumulated values of *LULI (ΣLULI)* as an estimate of the disease in the orchard.

**Figure 4:**
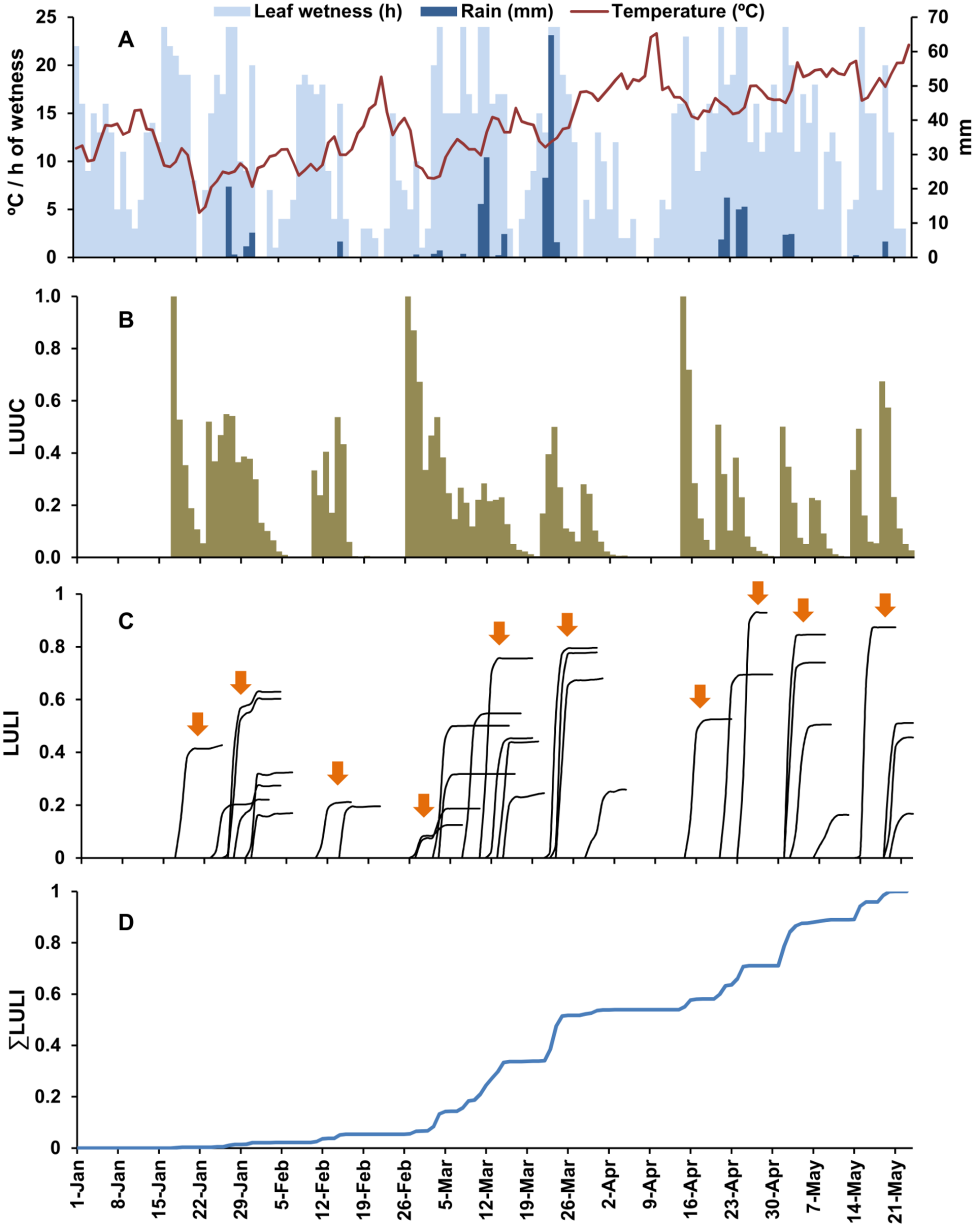
Weather data and model output in 2011. A: daily weather data; B: predicted frequency (%) of lesion units (LUs) with ungerminated conidia; C: predicted increase of LUs with latent infections (LULIs) for each infection period (arrows represent clusters of infection periods, clustering is based on an interval of at least 5 days between the beginning of two consecutive clusters); D: predicted seasonal dynamics of the cumulative values of LULI (ΣLULI).

**Figure 5:**
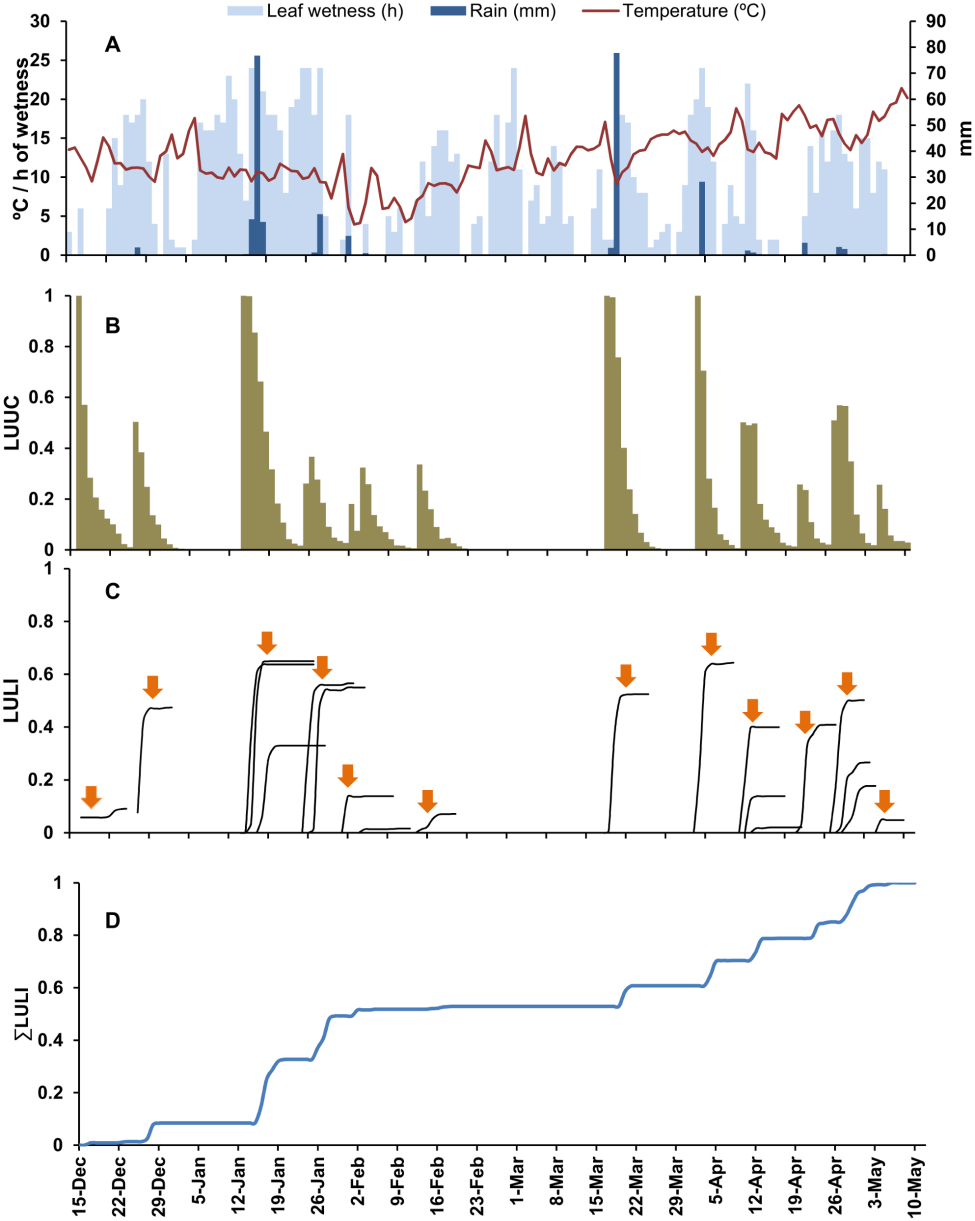
Weather data and model output in 2012. A: daily weather data; B: predicted frequency (%) of lesion units (LUs) with ungerminated conidia; C: predicted increase of LUs with latent infections (LULIs) for each infection period (arrows represent clusters of infection periods, clustering is based on an interval of at least 5 days between the beginning of two consecutive clusters); D: predicted seasonal dynamics of the cumulative values of LULI (ΣLULI).

Examples of model output for the 2011 and 2012 loquat growing seasons are shown in Figures 4 and 5, respectively. The output is based on the weather data registered by a weather station of the Regional Agrometerological Service (http://riegos.ivia.es/) located in Callosa d’En Sarrià, Alicante Province, southeastern Spain.

## Model Validation

Three data sets were used to validate the model: (i) incidence of affected fruits in a loquat orchard during growing seasons 2011 and 2012; (ii) disease occurrence on loquat fruits in single-exposure experiments in 2013; and (iii) expert assessment of the disease severity in seven loquat growing seasons.

To operate the model, hourly values of air temperature (T, °C), relative humidity (RH, %), and total rainfall (R, mm) were registered by the weather station of Callosa d’En Sarrià, which is ≤3.5 km from the orchards considered for validation.

### Predicted vs. observed disease incidence in orchards

In data set (i), observations were carried out in a loquat orchard in Callosa d’En Sarrià, Alicante Province, southeastern Spain. Details on these data have been previously published [7]. Briefly, fruits from four shoots of each of 46 loquat trees were assessed weekly, and disease incidence was expressed as the percentage of fruits with scab symptoms. The disease incidence was lower in 2011 than 2012, with 27.3% and 97.6% of fruits affected by loquat scab at harvest, respectively [7]. This difference in disease incidence may be related to the fact that the orchard was treated with fungicides for scab control in 2010 but not in 2011 or 2012. Given that the inoculum sources for fruit infection in 2011 was very low because of effective disease control in 2010, a correction factor for LUUC was applied for the infection processes initiated in January 2011, i.e., LUUC = 0.1 instead of  = 1 in January 2011.

Model validation was performed by comparing ΣLULI with observed data of disease incidence. Because there is a time lag (i.e., a latency period) between the predicted disease (as ΣLULI) and the disease incidence estimated in the orchard (DI), DI was shifted back by one latency period for comparison between predicted and observed disease. Sanchez-Torres et al. [1] observed a latency period of 21 days at a constant temperature of 20°C, which is a degree-day accumulation (DD base 0°C) of 420. Therefore, DI was shifted back by either 21 days or 420 DD. To calculate the DD, the average temperature of each day was considered with base temperature of 0°C.

### Predicted vs. observed disease incidence in single-exposure experiments

In data set (ii), data were collected in an abandoned loquat orchard in Callosa d’En Sarrià from 4 February to 15 April 2013. On 25 January, 200 random shoots bearing fruits were covered with water-resistant paper bags (one shoot per bag) to prevent deposition of rain-splashed conidia. On 4 February, 10 random bags were opened to receive splashed inoculum; after seven additional days, the bags were closed again. Ten other randomly selected bags were opened on 11 February and closed again 7 days later. This operation was repeated until nine groups of shoots had been sequentially exposed to rain. At the end of the experiment (15 April 2013), disease incidence (percentage of fruits affected by loquat scab) and severity were assessed in each group of shoots. Disease severity refers to the percentage of fruit area covered by scab lesions and was measured as described by González-Domínguez et al. [8].

Model validation was performed by comparing the model output in the week when a group of shoots was exposed to splashing rain with final disease severity in that group.

### Expert assessment

For data set (iii), Esteve Soler (technical advisor of the ‘Cooperativa Agricola de Callosa d’En Sarrià’) was asked to provide a subjective estimate of the severity (low, medium, or high) of loquat scab in the area for eight growing seasons (from 2005/2006 to 2012/2013). Mr. Soler’s estimates were based on his extensive experience in managing loquat orchards, on his scouting activities in the orchards of the cooperative, and on the number of fungicide treatments that were required to control the disease in the area.

For each season, the model was operated from 1 November to 31 March, and the numbers of disease outbreaks predicted by the model were counted. A disease outbreak was defined as *ΣLULI*>0.1 in 1 day, when no outbreaks were predicted in the previous 5 days. Average and standard error of the number of predicted outbreaks were calculated for each category (low, medium, or high) of scab severity derived from the expert assessment.

### Data analysis

Linear regression was used to compare the predicted and observed data of data sets (i) and (ii). To make data homogeneous, *ΣLULI* values at the time of each disease assessment in the orchards were rescaled to the *ΣLULI* at the end of the season; disease incidence was also rescaled to the final disease incidence. A *t*-test was used to test the null hypotheses that “a” (intercept of regression line) was equal to 0 and that “b” (slope of regression line) was equal to 1 [62]. The distribution of residuals of predicted versus observed values was examined to evaluate the goodness-of-fit. The concordance correlation coefficient (CCC) was calculated as a measure of model accuracy [63]; CCC is the product of two terms: the Pearson product-moment correlation coefficient between observed and predicted values and the coefficient Cb (bias estimation factor), which is an indication of the difference between the best fitting line and the perfect agreement line (CCC = 1 indicates perfect agreement). The following indexes of goodness-of-fit were also calculated [64]: NS model-efficacy coefficient, which is the ratio of the mean square error to the variance in the observed data, subtracted from unity (when the error is zero, NS = 1, and the provides a perfect fit); the W index of agreement which is the ratio between mean square error and total potential error (W = 1 represents a perfect fit); model efficiency (EF) which is a dimensionless coefficient that takes into account both the index of disagreement and the variance of the observed values (when EF increases toward 1, the fit increases); and the coefficient of residual mass (CRM) which is a measure of the tendency of the to overestimate or underestimate the observed values (a negative CRM indicates a tendency of the model toward overestimation).

For data set (iii), a one-way analysis of variance (ANOVA) was performed to determine whether the numbers of outbreaks predicted by the model in each category of loquat scab severity defined by the expert (i.e., low, medium, or high) were significantly different from one another.

## Results of Model Validation

### Predicted vs. observed disease incidence in orchards

In 2011 between 1 January (fruit set) and 23 May (harvest), 257.6 mm of rain fell, distributed in three main periods: the last week of January, the second week of March (with 64.8 mm of rain in 1 day), and the last 2 weeks of April (with daily temperature >15°C) (Fig. 4A). According to the model, a total of 33 infection periods were triggered by these rain events, and the first was on 17 January (Fig. 4B and 4C). In the analysis of this model output, infection periods were clustered in “infection clusters” based on an interval of a minimum of 5 days elapsed between the beginning of two consecutive infection clusters (i.e., the protection provided by a copper-based fungicide application as described in [65]); therefore, there were 10 infection clusters in the considered period. *ΣLULI* began to increase from mid-January to mid-February (with three infection clusters), but three infection clusters in March resulted in a substantial increase in *ΣLULI* to 0.5; March had 12 infections periods, and the repeated and abundant rain events provided >18 h of wetness on most days (Fig. 4). From mid-April to the end of the considered period, a constant increase in *ΣLULI* was associated with abundant rain events and increasing temperature, which triggered four infection clusters (Fig. 4).

In 2012, although the total volume of rain that fell from 15 December to 10 May was similar (255.8 mm) to that in 2011, there were fewer rain events. The model predicted 20 infection periods that were grouped into 12 infection clusters (Fig. 5B and 5C). In 2012, rainy periods were separated by dry periods; from the end of January to mid-March, dry periods caused no substantial infection to develop (Fig. 5C). Therefore, there were two main periods of *ΣLULI* increase: the last half of January and from the end of March to May (Fig. 5D).

**Figure 6:**
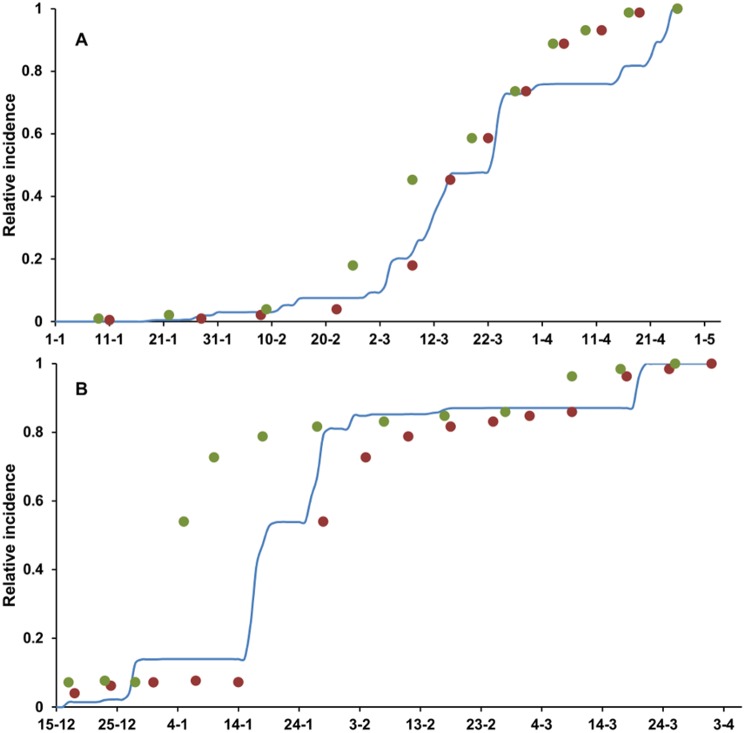
Comparison between model output and scab observed on loquat fruit in southeastern Spain. (A) data from 2011 and (B) data from 2012. Blue lines represent the rescaled infection predicted by the model as the seasonal summation of the lesion units with latent infections (ΣLULI). Points represent rescaled incidence of loquat fruit with scab observed in the orchards; rescaled incidence is shifted back by 21 days (red points) or 420 DD (base 0°C, green points) to account for the latency period, i.e., the time elapsed between infection and visible symptoms in the form of sporulating scab lesions.

Goodness-of-fit of predicted (*ΣLULI*) versus observed data (loquat scab incidence) was greater when a fixed period of 21 days was considered for the latency. In this case, values of R^2^, CCC, r, Cb, NS, W, and EF were >0.95 (Table 2). However, when a latency period of 420 DD was considered the values of R^2^ and CCC were <0.88, and values of model efficacy (NS) and model efficiency (EF) were 0.75 (Table 2) as a consequence of the high dispersion of residues in 2012 (Figure 6). The model slightly overestimated scab incidence when a latency of 21 days was used (CRM = −0.009) and underestimated scab incidence when DD were used (CRM = 0.182) (Table 2; Figure 6). For both latency options, the regression equations of predicted versus observed data had slopes and intercepts that were not significantly different from 1 and 0, respectively.

**Table 2:** Statistics and indices used for evaluating the goodness-of-fit of loquat scab infection predicted by the model versus disease observed in field.

Data set^a^	A^b^	b	*P*(a = 0)	*P*(b = 1)	*R^2^*	CCC	r	Cb	NS	W	EF	CRM
Data set 1(latency = 21 days)	0.038	0.939	0.190	0.070	0.952	0.974(0.946–0.988)	0.975	0.999	0.951	0.987	0.951	−0.009
Data set 1(latency = 420 DD)	−0.066	0.928	0.274	0.110	0.841	0.882(0.758–0.944)	0.921	0.960	0.753	0.939	0.752	0.182
Data set 2	0.043	0.965	0.02	0.1	0.984	0.986(0.942–0.996)	0.993	0.993	0.971	0.992	0.971	−0.247

a Data set 1 corresponds to comparison of daily accumulated LUVI predicted by the model versus observed data of loquat scab incidence in an orchard in southeastern Spain during 2 years (2011 and 2012). The model used a latency period of 21 days (first row) or 420 DD (second row). Data set 2 compares the increase of model output in weeks in which loquat shoots were exposed to splashing rain (triggering infection) with final disease severity in those shoots.

b a and b, parameters of the regression line of the predicted against observed values; *P*, probability level for the null hypotheses that a = 0 and b = 1; *R^2^,* coefficient of determination of the regression line; CCC, concordance correlation coefficient; r, Pearson product-moment correlation coefficient; Cb, bias estimation factor; NS, model efficacy; W, index of agreement; EF, model efficiency; CRM, coefficient of residual mass.

### Predicted vs. observed disease incidence in single-exposure experiments

**Figure 7:**
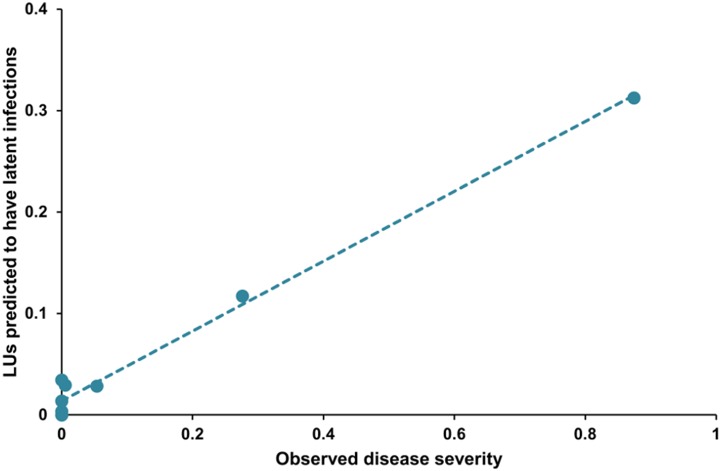
Comparison between model output and scab on loquat fruit in single-exposure experiments. Experiments were carried out in a loquat orchard in southeastern Spain in 2013. Observed data (X axis) are expressed as the rescaled disease severity in 11 groups of fruits that were exposed (for 7-day-long moving periods) to splashing rain in a severely affected orchard; model output (Y axis) is expressed as the summation of the lesion units with latent infections (ΣLULI) in the exposure period.

From 4 February to 15 April 2013, the model predicted 15 loquat scab infection periods but disease outbreaks were substantial (i.e., they resulted in a >10% increase in severity) in only two exposure periods. In these two cases, *LULI* values were >0.1; when there were no or light outbreaks, *LULI* values were <0.06 (Figure 7). The goodness-of-fit of predicted versus observed for data set (ii) (Table 2) provided values >0.97 for R^2^, CCC, r, Cb, NS, W, and EF. Although the slope was not significantly different from 1, the intercept was different from 0 at *P* = 0.02 (Table 2). The negative value of CRM indicated that the model somewhat overestimated disease, mainly when observed disease severity was low (Figure 7).

### Expert assessment

The loquat scab epidemics that occurred in the eight seasons of data set (iii) were considered by the expert to be of low, medium, or high severity in two, three, and three seasons, respectively. The number of outbreaks predicted by the model ranged from 4 to 17 among the eight seasons; in average, 8.5±0.5 outbreaks were predicted for years with low value of loquat scab severity, 10±3 for year with medium value and 12±2.9 for years with high value. Although the average number of outbreaks predicted by the model increased as the expert assessment of disease severity increased, the number of predicted epidemics did not significantly differ among the severity categories (*P* = 0.71).

## Discussion

In this work, a dynamic model was developed to predict infection of loquat fruits by conidia of *F. eriobotryae*. The model uses a mechanistic approach to describe the infection process [53], [66], [67]: the model splits the disease cycle of *F. eriobotryae* into different state variables, which change from one state to the following state based on rate variables or switches that depend on environmental conditions by means of mathematical equations. The mathematical equations were developed using published data on *F. eriobotryae* conidial dispersion patterns [7] and on *F. eriobotryae* growth, conidial germination, and infection under different environmental conditions [1], [6]. In the absence of precise information, assumptions were made based on available knowledge.

Model validation showed that the model correctly predicted the occurrence of infection periods and the severity of any infection period, as demonstrated by the goodness-of-fit for the data collected on fruits exposed to single rainy periods. Because the purpose of the model is to be part of a warning system for loquat scab management, the ability to correctly predict infection periods is crucial. Accuracy of the model was also confirmed by the comparison of model output with expert assessment. Even though the numbers of predicted outbreaks did not differ among seasons that the expert had categorized as having low, medium, or high disease severity, the number of predicted outbreaks increased with increases in assessed disease severity.

For model validation, the latency period required for the appearance of scab was expressed as a fixed number of days or of degree-days (DD) based on results from Sánchez-Torres et al. [1]. Goodness-of-fit of model prediction was overall better using a fixed period of 21 days instead of 420 DD. In particular, the model underestimated the disease in the early season of 2012 when DD were used. The underestimation was probably caused by low temperatures in that period, which delayed DD accumulation. This result is questionable, because the physiological development of fungi is usually more closely related to DD than to calendar days [68], [69]. In this work, DD was fixed based on the latency period observed in loquat plants kept at the optimal temperature for *F. eriobotryae* development, i.e., 21 days at 20°C [1]. Therefore, the DD value used in this study did not account for the non-linear response of *F. eriobotryae* growth to temperatures between 5 and 30°C [6]. If a function for predicting the appearance of scab symptoms is needed in the model, such a function should be temperature dependent, as it is in models for *V. nashicola* [37] and *F. oleagineum* [40]. Salerno et al. [4] repeatedly exposed potted loquat plants under the canopies of affected trees for 3 days and then incubated these plants under a roof until the appearance of symptoms. Scab appeared in 11 to 26 days at temperatures ranging from 11.4 to 17°C (with a DD range of 157 to 340) and after >220 days at temperatures >20°C. Ptskialadze [5] found scab symptoms on both leaves and fruits 34 and 16 days after infection at 1–4°C and 21–25°C, respectively. Even though the calculation of latency can be improved, the model error in predicting disease onset due to a fix latency period may not reduce the ability of the model to correctly predict infection periods or reduce the value of the model for timing fungicides applications.

The model capitalized on recent research concerning loquat scab [1], [6], [7]. These studies have considered most of the components of the disease cycle, including dispersion of conidia, infection, incubation, and latency. Nevertheless, other components should be elucidated to improve our knowledge and thus to improve the model [66]. Currently, the model assumes that inoculum sources are always present in scab-affected loquat orchards and that viable *F. eriobotryae* inoculum is always present at fruit set (i.e., when the model begins operating) and beyond. Salerno et al. [4] found that lesions appear in autumn on leaves that were infected the previous spring, and Prota [3] found that the lesions appearing in autumn produce conidia for 5 to 6 months and that those viable conidia are present all year long. These observations were carried out in Sicily and Sardinia, respectively (i.e., under a Mediterranean climate); therefore, the model assumptions seem plausible. The assumptions that inoculum sources and viable conidia are always present in scab-affected loquat orchards are both precautionary because they can lead to over prediction of infection (which would occur if weather conditions were suitable for infection but no viable conidia were available) and thus to unnecessary applications of fungicides or other disease management measures. Because unnecessary fungicide applications entail costs for growers, consumers, and the environment [51], the model should be expanded to include the oversummering and availability of conidia.

With respect to oversummering, modeling the dormant stage of fungal pathogens is challenging [66], and the dormant stage has therefore been included in only a few models [70]–[74]. For this purpose, two key aspects must be addressed: (i) the inoculum dose (i.e., the quantity of inoculum that oversummers), which depends on the severity of the disease in each orchard at the end of the previous season; and (ii) the time when the primary inoculum begins to be available for infection. In other models, the inoculum dose was directly measured in the field [70], [74] or broadly estimated as low/high disease pressure [72]. In our case, incorporation into the model of the specific farmer’s assessment of the disease severity in the previous season may represent useful information regarding the potential primary inoculum dose. Modeling the sporulation patterns of *F. eriobotryae* may make it possible to estimate the available inoculum at each infection period. This estimation may consequently improve the ability of the model to predict the severity of each infection period. To account for the presence of inoculum in a model for *V. inaequalis*, Xu et al. [39] assumed a minimum interval of 7 hours between two successive infection processes to allow lesions to recover and sporulate, even though this approximation could introduce errors, because sporulation is highly dependent on temperature and RH [14].

Even without the above possible improvements, the present model can contribute to the practical control of loquat scab. The underutilization of disease predictive systems by farmers have been broadly discussed [46], [47], [51], [75], [76]. Rossi et al. [53] summarized the steps necessary for the practical implementation of a model as: (i) develop a computerized version of the model; (ii) create a network of agro-meteorological stations for collecting weather data; (iii) design a strategy for decision-making based on the model output; (iv) develop tools for supporting decision-making (e.g., decision support systems or disease warning systems); and (v) build user’s confidence in the model by demonstrating the advantages of its use in comparison with the current options. Efforts devoted to the last three steps are crucial for the future applicability of the model [53] and requires a deep knowledge of the cultural context in which the model will be delivered, the farmers’ perception of risk, and the current management of the disease [47].

In the main loquat cultivation areas of Spain, the regional plant protection services use the Mills-Laplante tables [77], which were developed to control apple scab, to estimate the risk of infection by *F. eriobotryae* [78]. Researchers have indicated that the Mills-Laplante tables over-predict the number of infections for apple scab [37], [79]. That the tables could over-predict the number of loquat scab infections has also been discussed, because the conidia of *F. eriobotryae* require longer times for leaf infection than those described by the Mills-Laplante tables for *V. inaequalis* and because the temperature range in which *F. eriobotryae* infection occurs is quite different [6], [7]. Thus, the present model represents an improvement in loquat scab management, i.e., it should optimise scab management by helping loquat growers to schedule and probably to reduce fungicide applications.

The long-term existence of a warning system for loquat scab monitoring in Spain [80] may facilitate the implementation of the model developed in this area because i) extension agents and advisors are familiar with the use and interpretation of epidemiological models, and ii) loquat farmers are accustomed to considering the concept of “infection risk” when scheduling fungicide applications.

Because model building is “a never-ending story” [47], [81], researchers will likely continue to improve the loquat scab model described here. As discussed in this manuscript, it will be necessary to define a relationship between model output and infection severity so as to identify appropiate thresholds for deciding when the treatments are needed [53], [63].

## References

[1] Sánchez-Torres P, Hinarejos R, Tuset JJ (2009) Characterization and pathogenicity of *Fusicladium eriobotryae*, the fungal pathogen responsible for loquat scab. Plant Dis 93: 1151–1157. 10.1094/PDIS-93-11-1151 30754586

[2] Gladieux P, Caffier V, Devaux M, Le Cam B (2010) Host-specific differentiation among populations of *Venturia inaequalis* causing scab on apple, pyracantha and loquat. Fungal Genet Biol 47: 511–521. 20060485 10.1016/j.fgb.2009.12.007

[3] Prota U (1960) Ricerche sulla «ticchiolatura del Nespolo del Giappone e sul suo agente (*Fusicladium eriobotryae* Cav.). I. Observazioni sull’epidemiologia della malattia e sui caratteri morfo-biologici del parassita in Sardegna. Stud di Sassari 8: 175–196.

[4] Salerno M, Somma V, Rosciglione B (1971) Ricerche sull’epidemiologia della ticchiolatura del nespolo del Giappone. Tec Agric 23: 3–15.

[5] Ptskialadze L (1968) The causal agent of loquat scab and its biological characteristics. Rev Appl Mycol 47: 268.

[6] González-Domínguez E, Rossi V, Armengol J, García-Jiménez J (2013) Effect of environmental factors on mycelial growth and conidial germination of *Fusicladium eriobotryae*, and the infection of loquat leaves. Plant Dis 97: 1331–1338. 10.1094/PDIS-02-13-0131-RE 30722148

[7] González-Domínguez E, Rossi V, Michereff SJ, García-Jiménez J, Armengol J (2014) Dispersal of conidia of *Fusicladium eriobotryae* and spatial patterns of scab in loquat orchards in Spain. Eur J Plant Pathol 139: 849–861.

[8] González-Domínguez E, Martins RB, Del Ponte EM, Michereff SJ, García-Jiménez J, et al (2014) Development and validation of a standard area diagram set to aid assessment of severity of loquat scab on fruit. Eur J Plant Pathol 139: 413–422.

[9] Becker CM, Burr TJ (1994) Discontinuous wetting and survival of conidia of *Venturia inaequalis* on apple leaves. Phytopathology 84: 372–378.

[10] Hartman JRR, Parisi L, Bautrais P (1999) Effect of leaf wetness duration, temperature, and conidial inoculum dose on apple scab infections. Plant Dis 83: 531–534. 10.1094/PDIS.1999.83.6.531 30849828

[11] Holb IJ, Heijne B, Withagen JCM, Jeger MJ (2004) Dispersal of *Venturia inaequalis* ascospores and disease gradients from a defined inoculum source. J Phytopathol 152: 639–646.

[12] Rossi V, Giosue S, Bugiani R (2003) Influence of air temperature on the release of ascospores of *Venturia inaequalis* . J Phytopathol 151: 50–58.

[13] Stensvand A, Gadoury DM, Amundsen T, Semb L, Seem RC (1997) Ascospore release and infection of apple leaves by conidia and ascospores of *Venturia inaequalis* at low temperatures. Phytopathology 87: 1046–1053. 18945039 10.1094/PHYTO.1997.87.10.1046

[14] Machardy WE (1996) Apple scab. Biology, epidemiology and management. St. Paul: APS Press. 545.

[15] James J, Sutton TB (1982) Environmental factors influencing pseudothecial development and ascospore maturaion of *Venturia inaequalis* . Phytopathology 72: 1073–1080.

[16] Boric B (1985) Influence of temperature on germability of spores of *Venturia inaequalis* (Cooke) Winter, and their viability as affected by age. Zast Bilja 36: 295–302.

[17] Li B, Zhao H, Xu X-M (2003) Effects of temperature, relative humidity and duration of wetness period on germination and infection by conidia of the pear scab pathogen (*Venturia nashicola*). Plant Pathol 52: 546–552.

[18] Li BH, Xu XM, Li JT, Li BD (2005) Effects of temperature and continuous and interrupted wetness on the infection of pear leaves by conidia of *Venturia nashicola* . Plant Pathol 54: 357–363.

[19] Umemoto S (1990) Dispersion of ascospores and conidia of causal fungus of japanese pear scab, *Venturia nashicola* . Ann Phytopathol Japan Soc 56: 468–473.

[20] Rossi V, Salinari F, Pattori E, Giosuè S, Bugiani R (2009) Predicting the dynamics of ascospore maturation of *Venturia pirina* based on environmental factors. Phytopathology 99: 453–461. 19271988 10.1094/PHYTO-99-4-0453

[21] Spotts RA, Cervantes A (1991) Effect of temperature and wetness on infection of pear by *Venturia pirina* and the relationship between preharvest inoculation and storage scab. Plant Dis 75: 1204–1207.

[22] Spotts RA, Cervantes A, Cervantes LA (1994) Factors affecting maturation and release of ascospores of *Venturia pirina* in oregon. Phytopathology 84: 260–264.

[23] Villalta O, Washington WS, Rimmington GM, Taylor PA (2000) Influence of spore dose and interrupted wet periods on the development of pear scab caused by *Venturia pirina* on pear (*Pyrus communis*) seedlings. Australas Plant Pathol 29: 255–262.

[24] Villalta ON, Washington WS, Rimmington GM, Taylor PA (2000) Effects of temperature and leaf wetness duration on infection of pear leaves by *Venturia pirina* . Aust J Agric Res 51: 97–106.

[25] Lan Z, Scherm H (2003) Moisture sources in relation to conidial dissemination and infection by *Cladosporium carpophilum* within peach canopies. Phytopathology 93: 1581–1586. 18943623 10.1094/PHYTO.2003.93.12.1581

[26] Lawrence E, Zehr E (1982) Enviromental effects on the development and dissemination of *Cladosporium carpophilum* on peach. Phytopathology 72: 773–776.

[27] Gottwald TR, Bertrand PF (1982) Patterns of diurnal and seasonal airborne spore concentration of *Fusicladium effusum* and its impact on a pecan scab epidemic. Phytopathology 72: 330–335.

[28] Gottwald TR (1985) Influence of temperature, leaf wetness period, leaf age, and spore concentration on infection of pecan leaves by conidia of *Cladosporium caryigenum* . Phytopathology 75: 190–194.

[29] Latham AJ (1982) Effect of some weather factors and *Fusicladium effusum* conidium dispersal on pecan scab occurrence. Phytopathology 72: 1339–1345.

[30] De Marzo L, Frisullo S, Lops F, Rossi V (1993) Possible dissemination of *Spilocaea oleagina* conidia by insects (*Ectopsocus briggsi*). EPPO Bull 23: 389–391.

[31] Lops F, Frisullo S, Rossi V (1993) Studies on the spread of the olive scab pathogen, *Spilocaea oleagina* . EPPO Bull 23: 385–387.

[32] Obanor FO, Walter M, Jones EE, Jaspers MV (2008) Effect of temperature, relative humidity, leaf wetness and leaf age on *Spilocaea oleagina* conidium germination on olive leaves. Eur J Plant Pathol 120: 211–222.

[33] Obanor FO, Walter M, Jones EE, Jaspers MV (2010) Effects of temperature, inoculum concentration, leaf age, and continuous and interrupted wetness on infection of olive plants by *Spilocaea oleagina* . Plant Pathol 60: 190–199.

[34] Viruega JR, Moral J, Roca LF, Navarro N, Trapero A (2013) *Spilocaea oleagina* in olive groves of southern Spain: survival, inoculum production, and dispersal. Plant Dis 97: 1549–1556. 10.1094/PDIS-12-12-1206-RE 30716819

[35] Viruega JR, Roca LF, Moral J, Trapero A (2011) Factors affecting infection and disease development on olive leaves inoculated with *Fusicladium oleagineum* . Plant Dis 95: 1139–1146. 10.1094/PDIS-02-11-0126 30732070

[36] Eikemo H, Gadoury DM, Spotts RA, Villalta O, Creemers P, et al (2011) Evaluation of six models to estimate ascospore maturation in *Venturia pyrina* . Plant Dis 95: 279–284. 10.1094/PDIS-02-10-0125 30743503

[37] Li B, Yang J, Dong X, Li B, Xu X (2007) A dynamic model forecasting infection of pear leaves by conidia of *Venturia nashicola* and its evaluation in unsprayed orchards. Eur J Plant Pathol 118: 227–238.

[38] Rossi V, Bugiani R (2007) A-scab (Apple-scab), a simulation model for estimating risk of *Venturia inaequalis* primary infections. EPPO Bull 37: 300–308.

[39] Xu X, Butt DJ, Santen VAN (1995) A dynamic model simulating infection of apple leaves by *Venturia inaequalis* . Plant Pathol 44: 865–876.

[40] Roubal C, Regis S, Nicot PC (2013) Field models for the prediction of leaf infection and latent period of *Fusicladium oleagineum* on olive based on rain, temperature and relative humidity. Plant Pathol 62: 657–666.

[41] Payne AF, Smith DL (2012) Development and evaluation of two pecan scab prediction models. Plant Dis 96: 1358–1364. 10.1094/PDIS-03-11-0202-RE 30727191

[42] Trapman M, Jansonius PJ (2008) Disease management in organic apple orchards is more than applying the right product at the correct time. Ecofruit-13th International Conference on Cultivation Technique and Phytopathological Problems in Organic Fruit-Growing: Proceedings to the Conference from 18^th^ February to 20th February 2008 at Weinsberg/Germany. 16–22.

[43] Holb IJ, Jong PF, Heijne B (2003) Efficacy and phytotoxicity of lime sulphur in organic apple production. Ann Appl Biol 142: 225–233.

[44] Jamar L, Cavelier M, Lateur M (2010) Primary scab control using a “during-infection” spray timing and the effect on fruit quality and yield in organic apple production. 14: 423–439.

[45] Giosuè S, Bugiani R, Caffi T, Pradolesi GF, Melandri M, et al (2010) Used of the A-scab model for rational control of apple scab. IOBC WPRS Bull 54: 345–349.

[46] Rossi V, Caffi T, Salinari F (2012) Helping farmers face the increasing complexity of decision-making for crop protection. Phytopathol Mediterr 51: 457–479.

[47] Gent DH, Mahaffee WF, McRoberts N, Pfender WF (2013) The use and role of predictive systems in disease management. Annu Rev Phytopathol 51: 267–289. 23682914 10.1146/annurev-phyto-082712-102356

[48] Boller EEF, Avilla J, Gendrier JP, Jörg E, Malavolta C (1998) Integrated Production in Europe: 20 years after the declaration of Ovronnaz. IOBC Bull 21: 1–34.

[49] Alavanja MCR, Hoppin JA, Kamel F (2004) Health effects of chronic pesticide exposure: cancer and neurotoxicity. Annu Rev Public Health 25: 155–197. 15015917 10.1146/annurev.publhealth.25.101802.123020

[50] Brent KJ, Hollomon DW (2007) Fungicide resistance in crop pathogens: How can it be managed? FRAC Monog 2. Fungicide Resistance Action Committee.

[51] Shtienberg D (2013) Will decision-support systems be widely used for the management of plant diseases? Annu Rev Phytopathol 51: 1–16. 23767845 10.1146/annurev-phyto-082712-102244

[52] Leffelaar P (1993) On Systems Analysis and Simulation of Ecological Processes. Kluwer. London.

[53] Rossi V, Giosuè S, Caffi T (2010) Modelling plant diseases for decision making in crop protection. In: Oerke E-C, Gerhards R, Menz G, Sikora RA, editors. Precision Crop Protection-the Challenge and Use of Heterogeneity.

[54] Hui C (2006) Carrying capacity, population equilibrium, and environment’s maximal load. Ecol Modell 192: 317–320.

[55] Townsend C, Begon M, Harper J (2008) Essentials of ecology. John Wiley and Sons. New York. 510.

[56] Zadoks J, Schein R (1979) Epidemiology and plant disease management. Oxford University Press, New York. 427.

[57] Bennett JC, Diggle A, Evans F, Renton M (2012) Assessing eradication strategies for rain-splashed and wind-dispersed crop diseases. Pest Manag Sci 69: 955–963. 10.1002/ps.3459 23355345

[58] Caffi T, Rossi V, Bugiani R, Spanna F, Flamini L, et al (2009) A model predicting primary infections of *Plasmopara viticola* in different grapevine-growing areas of Italy. J Plant Pathol 91: 535–548.

[59] Ghanbarnia K, Dilantha Fernando WG, Crow G (2009) Developing rainfall- and temperature-based models to describe infection of canola under field conditions caused by pycnidiospores of *Leptosphaeria maculans* . Phytopathology 99: 879–886. 19522586 10.1094/PHYTO-99-7-0879

[60] Gilligan CA, van den Bosch F (2008) Epidemiological models for invasion and persistence of pathogens. Annu Rev Phytopathol 46: 385–418. 18680429 10.1146/annurev.phyto.45.062806.094357

[61] Buck AL (1981) New equations for computing vapor pressure and enhancement factor. J Appl Meteorol 20: 1527–1532.

[62] Teng P (1981) Validation of computer models of plant disease epidemics: a review of philosophy and methodology. J Plant Dis Prot 88: 49–63.

[63] Madden L V, Hughes G, van den Bosch F (2007) The study of plant disease epidemics. APS press. St. Paul. 421.

[64] Nash J, Sutcliffe J (1970) River flow forecasting through conceptual models part I. J Hidrol. 10: 282–290.

[65] González-Domínguez E, Rodríguez-Reina J, García-Jiménez J, Armengol J (2014) Evaluation of fungicides to control loquat scab caused by *Fusicladium eriobotryae*. Plant Heal Prog Accepted.

[66] De Wolf ED, Isard SA (2007) Disease cycle approach to plant disease prediction. Annu Rev Phytopathol 45: 203–220. 17408356 10.1146/annurev.phyto.44.070505.143329

[67] Krause RA, Massie LB (1975) Predictive systems: modern approaches to disease control. Annu Rev Phytopathol 13: 31–47.

[68] Fourie P, Schutte T, Serfontein S, Swart F (2013) Modeling the effect of temperature and wetness on *Guignardia pseudothecium* maturation and ascospore release in citrus orchards. Phytopathology 103: 281–292. 23234366 10.1094/PHYTO-07-11-0194

[69] Gadoury DM, Machardy WE (1982) A model to estimate the maturity of ascospores of *Venturia inaequalis* . Phytopathology 72: 901–904.

[70] Holtslag QA, Remphrey WR, Fernando WGD, Ash GHB (2004) The development of a dynamic disease- forecasting model to control *Entomosporium mespili* on Amelanchier alnifolia. Can J Plant Pathol 313: 304–313.

[71] Legler SEE, Caffi T, Rossi V (2013) A Model for the development of Erysiphe necator chasmothecia in vineyards. Plant Pathol. DOI:10.1111/ppa.12145.

[72] Luo Y, Michailides TJ (2001) Risk analysis for latent infection of prune by *Monilinia fructicola* in California. Phytopathology 91: 1197–1208. 18943335 10.1094/PHYTO.2001.91.12.1197

[73] Rossi V, Caffi T, Giosuè S, Girometta B, Bugiani R, et al (2005) Elaboration and validation of a dynamic model for primary infections of *Plasmopara viticola* in North Italy. Riv Ital di Agrometeorol 13: 7–13.

[74] Gadoury D, Machardy WE (1986) Forecasting ascospore dose of *Venturia inaequalis* in commercial apple orchards. Phytopathology 76: 112–118.

[75] Gent DH, De Wolf E, Pethybridge SJ (2011) Perceptions of risk, risk aversion, and barriers to adoption of decision support systems and integrated pest management: an introduction. Phytopathology 101: 640–643. 21117876 10.1094/PHYTO-04-10-0124

[76] Schut M, Rodenburg J, Klerkx L, van Ast A, Bastiaans L (2014) Systems approaches to innovation in crop protection. A systematic literature review. Crop Prot 56: 98–108.

[77] Mills W, Laplante A (1954) Diseases and insect in the orchard. Cornell Ext Bull 711.

[78] GVA (2013) Octubre-Noviembre 2013. Butlletí d’avisos 13.

[79] Machardy WE, Gadoury DM (1989) A revisions of Mills’s criteria for predicting apple scab infection periods. Phytopathology 79: 304–310.

[80] González-Domínguez E, Armengol J, García-Jiménez J, Soler E (2013) El moteado del níspero en la Marina Baixa. Phytoma 247: 50–52.

[81] Teng P (1981) Validation of computer models of plant disease epidemics: a review of philosophy and methodology. J Plant Dis Prot 88: 49–63.

